# Rhamnolipids From *Pseudomonas aeruginosa* Are Elicitors Triggering *Brassica napus* Protection Against *Botrytis cinerea* Without Physiological Disorders

**DOI:** 10.3389/fpls.2018.01170

**Published:** 2018-08-08

**Authors:** Noadya Monnier, Aurélien Furlan, Camille Botcazon, Abdellatif Dahi, Gaëlle Mongelard, Sylvain Cordelier, Christophe Clément, Stéphan Dorey, Catherine Sarazin, Sonia Rippa

**Affiliations:** ^1^Unité de Génie Enzymatique et Cellulaire, CNRS UMR 7025, SFR Condorcet FR CNRS 3417, Université de Picardie Jules Verne, Amiens, France; ^2^Unité de Génie Enzymatique et Cellulaire, CNRS UMR 7025, SFR Condorcet FR CNRS 3417, Université de Technologie de Compiègne, Sorbonne Universités, Compiègne, France; ^3^Centre de Ressources Régional en Biologie Moléculaire, SFR Condorcet FR CNRS 3417, Université de Picardie Jules Verne, Amiens, France; ^4^Unité RIBP-EA 2069, SFR Condorcet FR CNRS 3417, Université de Reims Champagne Ardenne, Reims, France

**Keywords:** Rhamnolipids, *Brassica napus*, elicitor, defense responses, plant immunity, biocontrol

## Abstract

Rhamnolipids (RLs) are amphiphilic molecules naturally produced by some bacteria with a large range of biological activities. Although some studies report their potential interest in plant protection, evaluation of their effects and efficiency on annual crops of worldwide agronomic interest is lacking. The main objective of this work was to investigate their elicitor and protective activities on rapeseed crop species while evaluating their physiological effects. Here we report that RLs from *Pseudomonas aeruginosa* secretome trigger an effective protection of *Brassica* *napus* foliar tissues toward the fungus *Botrytis cinerea* involving the combination of plant defense activation and direct antimicrobial properties. We demonstrated their ability to activate canonical *B.* *napus* defense responses including reactive oxygen species production, expression of defense genes, along with callose deposits and stomatal closure as efficient physical protections. In addition, microscopic cell death observations and electrolyte leakage measurements indicated that RLs trigger a hypersensitive response-like defense in this plant. We also showed that foliar spray applications of RLs do not induce deleterious physiological consequences on plant growth or chlorophyll content and that RL protective properties are efficient on several grown cultivars of rapeseed. To our knowledge, this is the first report of RLs as an elicitor that suppresses fungal disease on tissues of an annual crop species under greenhouse conditions. Our results highlight the dual mode of action of these molecules exhibiting plant protection activation and antifungal activities and demonstrate their potential for crop cultures as environmental-friendly biocontrol solution.

## Introduction

Plants have developed potent defense mechanisms to counter act devastating microbial pathogens. Plant defense systems against phytopathogens can be constitutive or induced. Constitutive resistance includes preformed cuticular waxes, tough cell wall and phytoanticipins (Burketová et al., 2015; Pedras and Yaya, 2015). Induced resistance is triggered upon perception by the plant of Invasion Patterns (IPs) originating from the microorganisms or damaged plant tissues (Cook et al., 2015). This IP-triggered resistance is activated through multipurpose intracellular signaling that initiates the synthesis of metabolites and macromolecules. They are characterized by early responses (such as an oxidative burst, kinase phosphorylations, and early transcriptional changes) and late responses (such as late transcriptional changes, and callose deposits) (Bigeard et al., 2015; Cook et al., 2015).

Considering the global oilseed production, rapeseed (*Brassica napus* L.) is in second place just behind soybean in the world, and in first position in Europe (Fischer et al., 2014). Rapeseed is exposed to many fungal pathogens severely impacting agricultural yields (Verma et al., 2012). As a consequence, farmers use intensively preventive chemipesticides, mainly applied by foliar spraying, which have deleterious effects on health and environment (Lu, 2003). Since rapeseed is essentially cultivated for its oil, used for human nutrition purposes, and as a renewable source for fuels and technical oils, the sustainability of the raw plant material is of great importance (Kumar et al., 2016). Given the significance of cultivated areas, the development of biocontrol strategies to reduce the use of chemical pesticides is an essential alternative for the protection of this crop.

Rhamnolipids (RLs) are glycolipids produced by bacteria of the genera *Pseudomonas* and *Burkholderia.* These amphiphilic compounds are composed of one or two rhamnose glycosyl polar heads linked through a beta-glycosidic bond to one or two 3-hydroxyfatty acid hydrophobic tails (Abbasi et al., 2012). In bacteria, they are involved in surface motility, biofilm development and the uptake and degradation of poorly soluble substrates (Abdel-Mawgoud et al., 2010). RLs are well known as biosurfactant used for a wide range of industrial applications, especially in food, cosmetics, and pharmaceutical formulations, as well as in bioremediation of pollutants (Rikalovic et al., 2015).

For years, RLs have been extensively studied for their direct antimicrobial activities as reviewed in Vatsa et al., 2010. They were first described as having properties against gram-positive and gram-negative bacteria. They were also shown to possess antifungal activities toward oomycetes, ascomycetes and zygomycetes. Plant defense eliciting activities have been reported only recently in grapevine (Varnier et al., 2009) and *Arabidopsis thaliana* (Sanchez et al., 2012). RLs were also shown to trigger resistance of cherry tomato fruits toward *Alternaria alternata* (Yan et al., 2016). Due to their antimicrobial and defense eliciting properties, RLs are likely to possess potential applications in agriculture (Chen et al., 2017). Reducing production costs by using raw materials such as industrial waste or byproducts demonstrate the economic potential of these compounds (Bagheri Lotfabad et al., 2017; Chen et al., 2017). In addition, their low toxicity and biodegradability assets reinforce their attractiveness (Johann et al., 2016; Liu et al., 2016). However, studies evaluating RL effectiveness on annual crop plants and assessing their impact on plant physiology are missing.

In order to consider RLs as a part of biocontrol strategies, the objective of this study was to investigate the ability of RLs to set up rapeseed protection and canonical defense responses against the opportunistic phytopathogenic fungus *Botrytis cinerea*, the causal agent of gray mold, used here as a model. The effects were studied at molecular and physiological levels.

## Materials and Methods

### Biological Materials and Culture Conditions

*Brassica napus* cultivar Darmor-*bzh* seeds were obtained from the BrACySol Resource Center (INRA, Rennes, France). Atenzo and Gaspard cultivars were kindly provided by Limagrain (Saint-Beauzire, France) and SERASEM (Paris, France), respectively. Plants were cultivated in soil in controlled conditions (18°C, 16-h photoperiod, 400 μmol m^−2^ s^−1^ light intensity, 70% relative humidity). For *in vitro* grown seedlings, seeds were first surface sterilized 4 min in 70% ethanol and 15 min in 25% commercial bleach supplemented with 0.01% Tween 20 v/v. Seedlings were grown at 21°C, 60% relative humidity, 150 μmol m^−2^ s^−1^ light intensity with a 12-h photoperiod in plant growth medium [Murashige & Skoog basal medium (Sigma–Aldrich, St. Louis, MO, United States), 0.5 g L^−1^ MES (2-(*N*-morpholino)ethanesulfonic acid), and 5 g L^−1^ sucrose] solidified with agar (7 g L^−1^) and equilibrated to pH 5.7.

*Botrytis cinerea* strain B630 (INRA, Versailles, France) was cultivated by transferring a piece of agar containing mycelium on potato dextrose agar (Sigma–Aldrich), incubated at 18°C with a 16-h photoperiod. Conidia were collected with sterile water (2 mL) from 4-week-old culture plates and diluted to reach the appropriate concentration after counting with a Fuch-Rosenthal chamber.

### Treatment Solution Preparations and Analyzes

The RLs used in this study were a mix of 40% of α-L-rhamnopyranosyl-β-hydroxydecanoyl-β-hydroxy-decanoate (monoRLs) and 60% of 2-*O*-α-L-rhamnopyranosyl-β-L-rhamnopyr-anosyl-β-hydroxydecanoyl-β-hydroxydecanoate (diRLs) from *Pseudomonas aeruginosa* secretome (Varnier et al., 2009). Based on this composition with an average molecular weight of 591.6 g mol^−1^, a 0.6 g mL^−1^ solution was calculated to 1 M RL solution. A 11.8 μg mL^−1^ RL stock solution (ca. 20 mM) was prepared in 100% ethanol and diluted in water before treatments. According to sensitivity constraints of the different experiments, and taking into account the practical feasibility for applications, the two RL concentrations, 0.006 mg mL^−1^ (ca. 10 μM) and 0.06 mg mL^−1^ (ca. 100 μM), were used. Final ethanol concentration in RL treatment solutions was always lower or equal to 0.5% v/v with the same amount of ethanol in control and RL treatment solutions. The peptide flg22 (QRLSTGSRINSAKDDAAGLQIA) was synthesized by Proteogenix (Schiltigheim, France). The stock was diluted in water at 1 mM.

The scattered intensity in function of RL concentration was studied by dynamic light scattering measurements at 25.0 ± 0.3 °C with a laser emitting at 660 nm (DynaProNanostar, Wyatt Technology, Santa Barbara, CA, United States) to evaluate their aggregation behavior (Rikalovic et al., 2015). Measurements were carried out using a Dynamics 7.1.7 software (Wyatt Technology). 30–200 acquisitions were performed with an acquisition time ranging from 5 to 10 s according to the sample. The different auto-correlation functions obtained were fitted by a Dynals algorithm. Experiments were replicated three times to ensure reproducibility.

### Measurement of Reactive Oxygen Intermediate Production

Measurements of reactive oxygen species were performed on half 9-mm disks prepared with a cork-borer from leaves of 4-week-old plants using a method adapted from Smith and Heese (2014). The day before the experiment, disks were placed in water in a 96 wells plate. The day of measurements, the water was replaced by 180 μL of 20 μg mL^−1^ horseradish peroxidase and 0.2 μM luminol (Sigma-Aldrich). After 6 min, 20 μL of treatment solutions were added through injectors of a luminometer (TECAN Infinite^®^ M1000, Männedorf, Switzerland). Luminescence was monitored for 2 h with relative light unit (RLU) measurements every 2 min. Data corresponded to a mean of four independent biological repetitions obtained from six foliar disks.

### RNA Extraction and qRT-PCR Analyzes

For gene expression quantifications, three 2-day-old *in vitro* grown seedlings per condition were transferred to 950 μL of liquid growth medium the day before treatment. The next day, 50 μL of treatment solution were added to the medium until harvest time. RNA extractions were proceeded with the RNeasy plant mini kit (Qiagen, Hilden, Germany). RNA concentrations and qualities were determined and checked with a NanoDrop 1000 spectrophotometer (ThermoFisher Scientific, Waltham, MA, United States) and a 2100 Bioanalyzer (Agilent Technologies, Santa Clara, CA, United States). Reverse transcriptions were performed using MMuLV enzyme (ThermoFisher) on 4 μg RNA. Quantitative PCR were carried out using SYBR green master mix and a LightCycler^®^ 480 (Roche, Bâle, Switzerland) according to Gutierrez et al. (2012). Reference gene *BnELF5* was chosen for its stability in our conditions among seven genes tested using the GeNorm software (Vandesompele et al., 2002). F (Forward) and R (Reverse) primer sequences in 5′ to 3′ orientation were: F GGACTCATCGTTTGGTTCTTCTTTT R CGTTTTGGTTACGCTATGAACAGTC for *BnWRKY33*, F GAGATGTGGTTCCTCCACCA R ACTTGAGCCCTCGAAGAAGC for *BnMPK3* and F GCCAATGTGTTACACCGAGATC R CCGAAATCCCCAAGCTTTAGA for *BnMPK4*; (Lloyd et al., 2014) F ATGCCAACGCTCACAACCA R CACGGGACCTACGCCTACT for *BnPR1*, F CATCACCCTTCTCTTCGCTGC R ATGTCCCACTTGACCTCTCGC for*BnPDF1.2* (Wang et al., 2009); F TGGACCACCACCTATGATGA R GCTGCGGATTCTCTTCTGAC for *EFL5* (Yang et al., 2014); F CTTGGTTTCGATGAC GCAGAGC R CGACGTTGGTGCTGGAGATTTAC for *BnMYC2* (Aliakbari and Razi, 2013); F CACCGCTCCGTGAAGTTAGATA R ACCCCAAAAGCTCCTCAAGGTA for *BnERF1* (Babula-Skowrońska et al., 2015) and F GATTTGAGAGCCGTGAGTGC R CATTGCTGCATTGGTCCACA for *BnPR4*. The two last primers were designed using Primer3web© version 4.0.0 (Untergasser et al., 2012). All primers were synthetized by Eurofins Genomics (Luxembourg). Results were obtained from three biologically independent replicates. CT (crossing threshold) and PCR efficiency (E) values were used to calculate expression using the formula E_T_^(CT^_C_^−^^CT^_RL_^)^/E_R_^(CT^_C_^−^^CT^_RL_^)^, where T was the target gene and R was the reference gene, C refered to cDNA from the control plants and RL refered to cDNA from the treated plants. The relative expression corresponded to the value obtained for RL-treated plants relative to the value obtained for control plants.

### Stomatal Aperture Measurements

Foliar disks (7 mm in diameter) from 3 to 4-week-old plants were prepared with a cork-borer and immersed in RL or control solutions. After 3 h, disks were placed on glass slides and observed under a scanning confocal microscope (Carl Zeiss, Oberkochen, Germany). Auto-fluorescence of chlorophyll was observed using laser emissions at 638 and 488 nm. The images were optimized by scanning in XY mode while adjusting laser transmissivity and voltage of photomultiplier tubes. Then, in XYZ mode, Z-scans were performed. Three-dimensional reconstructions of images were done using ZEN 2.3 SP1 software (Carl Zeiss). The width of the stomatal aperture was measured using ImageJ software^1^. Aperture corresponded to the minor axis of an ellipse fitted on each stoma. A total of at least 140 stomata from 10 disks of 10 different plants per condition were used for average and standard error determinations.

### Callose Deposition Colorations

Ten-day-old to fifteen-day-old *B. napus* plants (two leaves stage) were sprayed with treatment solutions (1 mL for each plant). Leaves were harvested after 24 h and submerged into a 1/3 acetic acid/ethanol (v/v) solution for 12 h. Leaves were rinsed 30 min in 150 mM K_2_HPO_4_ before being transferred for 12 h in a 0.01% methyl blue (w/v) solution in the same buffer. Tainted leaves were embedded in 50% glycerol and observed with an epifluorescence microscope Leica DMI 6000B (Leica, Wetzlar, Germany) with a DAPI filter. Callose was quantified from photographs using ImageJ software as the number of callose corresponding pixels relative to the total number of pixels covering plant material. Contrast settings of the photographs were adjusted to obtain an optimal separation of the callose signal from the background signal. At least, 25 photographs from three independent experiments were used for average and standard error determinations.

### Electrolyte Leakage and Cell Viability Assessments

Foliar disks (7 mm in diameter, 4 per condition) from different 3-week-old plants were immersed in 3 mL of deionized water over night in 12 wells plate. The next day, water was replaced by 1 mL of RL or control solutions. The conductivity was measured over time on 100 μL with a conductimeter LAQUAtwin B-771 (Horiba, Kyoto, Japan). The solution was thereafter returned to the cell plate. Conductivity values were determined as the difference to initial measurement and were obtained from three biologically independent replicates. Cell viability measurements were performed with Evans blue method from Vijayaraghavareddy et al. (2017). After treatment, disks were stained 30 min in 0.25% (w/v) Evans blue, 0.1 M CaCl_2_ solution under stirring, then rinsed 3 times with 0.1 M CaCl_2,_ pH 5.6, and observed under light microscopy. For quantification, the wounded periphery of disks was removed by cutting a 6 mm disk from each original one. Number of colored cells were reported to the disk surfaces on four leaf disks per condition. Results corresponded to averages and standard errors from three biologically independent repetitions.

### Plant Protection Assays and Aniline Blue Colorations

Foliar disks (36 per condition) of 12 mm diameter from 4-week-old plants were submerged 30 min into RLs or control solution. The disks were briefly vacuum infiltrated to maintain the solutions on hydrophobic leaf surfaces (this was particularly necessary for the control disks without surfactant RLs). *B. cinerea* conidia were diluted to a 10^6^ mL^−1^ spore solution. Disks were then disposed in petri dishes on a wet filter paper, slightly wounded in the middle with a tip and inoculated with 5 μl of conidia. Lesions (one per disk) were observed with a binocular microscope 96 and 192 h after treatment. They were classified according to their area measurement. Results were expressed in percentage of foliar disks developing each category of lesions. Data were from one representative experiment among three independent repetitions. Aniline blue colorations of mycelium were adapted from Grace and Stribley (1991) and Davidson et al. (2016). Foliar disks were fixed and stored in 1/3 lactoglycerol (10 mL lactic acid/20 mL glycerol/10 mL water)/96% ethanol (v/v). Before observations, disks were placed in 1/3 acetic acid/96% ethanol (v/v) until total discoloration. The tissues were stained by transferring to 1/2 lactoglycerol blue (50 mg methyl blue in 40 mL lactoglycerol)/96 % ethanol (v/v) for 1 min. The disks were then rinsed and observed in 50% glycerol (v/v).

### *B. cinerea* Conidia Germination and Mycelium Growth Inhibition Determinations

For germination assays, 1.3 10^6^ mL^−1^ conidia were cultivated in liquid potato dextrose broth medium (Sigma-Aldrich) without shaking at 21°C in the dark. Germ tube appearing was counted under a light microscope over time after RL addition. For mycelium growth inhibition assays, 5.5 mm diameter mycelium plugs from *B. cinerea* were excised from V8 agar medium (16% V8 juice v/v, 2.4 g L^−1^ CaCO_3_, 16 g L^−1^ agar) and transferred to the center of new Petri dishes (diameter 9 cm) containing 0.06 mg mL^−1^ RLs or not. For each plate, mycelium radial growth was determined by measuring two perpendicular diameters. The values corresponded to the average of measurements from three independent plates for each condition. For each plate, the percent of growth inhibition (I) corresponded to the formula:

$$I=\frac{100(G_{\mathrm{RC}}-G_{\mathrm{RL}})}{G_{\mathrm{RC}}}$$

Gr_C_ related to the growth of the control and Gr_RL_ the growth of the RL treated plates. Measurements were stopped after 144 h to avoid side effects.

### Seedling Growth and Chlorophyll Content Measurements

For *in vitro* growth inhibition tests, 2-day-old seedlings were transferred to petri dishes containing agar growth medium supplemented with RL or control solutions. Length measurements and studies of fresh weight were done on day 7. Values corresponded to the average of measurements from three independent experiments (24 plants per condition). For quantifying spray effect, 3-day-old *in vitro* grown seedlings were transferred to a hydroponic culture system (Araponics, Liege, Belgium) containing liquid growth medium. On day 4, the aerial part of seedlings was sprayed until flow with RL solution. Pictures were taken on day 10 to allow root length measurement with ImageJ software. Aerial part and roots were weighted separately as fresh material and lyophilised over night before being weighted as dry material. For chlorophyll content quantification, samples were prepared by extraction of homogenized fresh aerial parts in 80% acetone (v/v) at laboratory temperature and analyzed at wavelengths 663, 646, and 470 nm on a spectrophotometer Cary 60 UV-Vis (Agilent Technologies). Concentrations were determined according to Wellburn (1994) and corresponded to the mean of three independent experiments (18 plants per condition).

### Statistical Analyses

Statistical differences in the plant tissue protection assays, seedling growth and chlorophyll content measurements were analyzed with Kruskal–Wallis test (*P* < 0.01). One-way ANOVA tests were performed for qRT-PCR analyzes (α = 0.05), stomatal aperture and callose deposit measurements (α = 0.01). Distinct groups were designated by different letters.

## Results

Before experiments on plants, the aggregation state of RLs was analyzed at 25°C by dynamic light scattering measurements. A critical aggregation concentration of ca. 10 μM (0.006 ± 0.001 mg mL^−1^) was obtained (**Supplementary Figure S1A**). Plant protective effects of RLs were previously observed from the hundred micromolar range (Varnier et al., 2009; Sanchez et al., 2012). The RL solutions used in this study were 0.006 mg mL^−1^ (ca. 10 μM) and 0.06 mg mL^−1^ (ca. 100 μM). At both concentrations, RLs were under aggregation state with average diameters of mainly 120 nm and 200 nm respectively. Aggregates of 40 nm and 55 nm were also present in a lesser extend in both solutions respectively (**Supplementary Figure S1B**).

**FIGURE 1:**
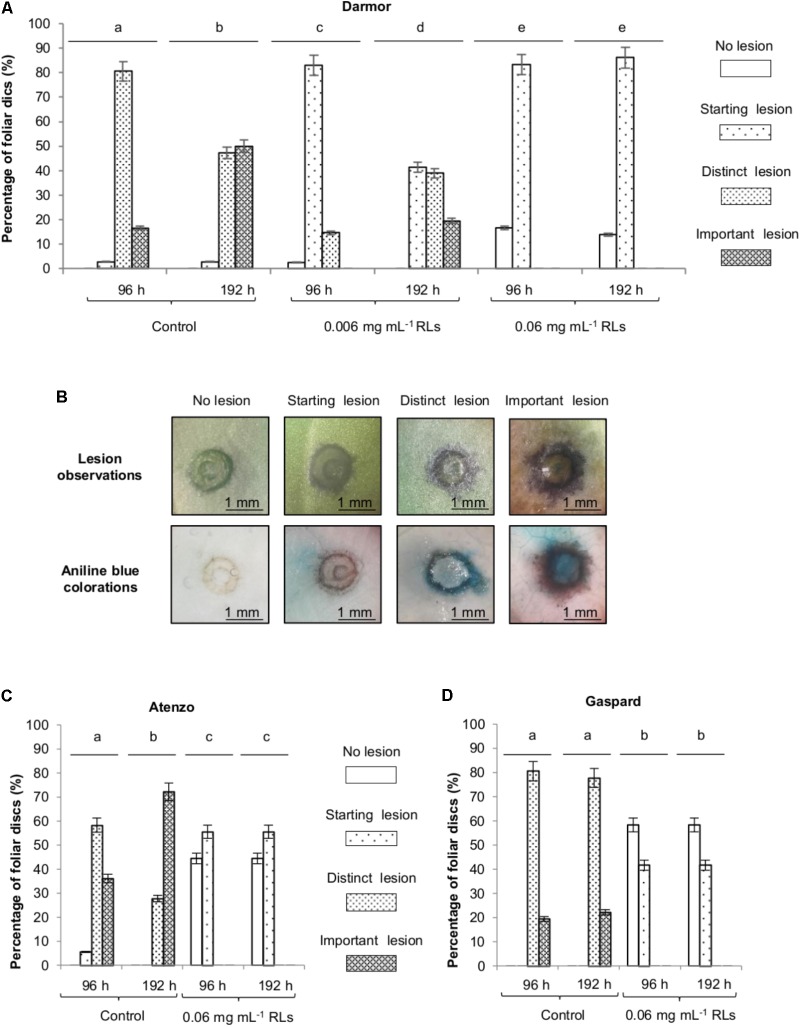
Rhamnolipids protection of *B. napus* foliar disks toward *B. cinerea.* **(A)** Effect of RLs on the development of *B. cinerea* on foliar disks of *B. napus* Darmor-*bzh*. Lesions were classified according to their area: no lesion, starting lesion (<1 mm^2^), distinct lesion (<2 mm^2^) and important lesion (>2 mm^2^). **(B)** Lesions were observed by a binocular loupe before and after aniline blue colorations. Effect of RLs (0.06 mg mL^−1^) on the development of *B. cinerea* on foliar disks of *B. napus* Atenzo **(C)** and Gaspard **(D)** cultivars. Distinct groups are designated by different letters (Kruskal–Wallis test, *P* < 0.01).

### RL Protection of *B. napus* Foliar Discs Against *B. cinerea*

In order to evaluate the potential of RLs to efficiently protect *B. napus* against the opportunistic pathogen *B. cinerea*, leaf tissue infections were performed. Foliar disks from 3 to 4 week-old plants of the sequenced reference cultivar *B. napus* Darmor-*bzh* (Chalhoub et al., 2014) were first submerged in 0.006 mg mL^−1^, 0.06 mg mL^−1^ RL or control solutions and then inoculated with conidia. After 96 h, all the non-RL-treated foliar disks presented symptoms due to pathogen infections with different size areas: (i) starting lesions, less than 1 mm^2^, appeared on 3%, (ii) distinct lesions, between 1 and 2 mm^2^, on 80%, (iii) important lesions on 17% of the disks (**Figures 1A,B**). Mycelium staining with aniline blue confirmed the size of the lesions observed (**Figure 1B**). Ninety seven percent of the 0.006 mg mL^−1^ RL-treated disks presented symptoms after 96 h with 83% of starting lesions and 14% of distinct lesions but no important lesions. On the contrary, 17% of the 0.06 mg mL^−1^ RL-treated disks did not present any symptoms and, the remaining 83% developed only starting lesions (**Figure 1A**). No distinct or important lesions (more than 2 mm^2^) were observed on the 0.06 mg mL^−1^ RL-treated foliar disks after 192 h, whereas they represented 97% of the control foliar disks and 60% of the 0.006 mg mL^−1^ RL-treated foliar disks. A very limited evolution of lesions was visible on the 0.06 mg mL^−1^ RL-treated disks between 96 and 192 h showing the durability of the RL effect in these conditions. Aniline blue staining clearly showed a decrease of mycelium development (**Supplementary Figure S2**). Hence, a relevant number reduction of *B. cinerea* pathogenic lesions was observed after a pretreatment of foliar disks with RLs as compared to non-pretreated disks. Their size were decreased and their evolution slowed down in a concentration-dependent manner.

As responses to different IPs are known to be genotype dependent (Lloyd et al., 2014), protection assays were performed on Atenzo (oleaginous) and Gaspard (erucic) cultivars, grown for alimentary and industrial purposes, respectively. After 96 h, all non-RL-treated foliar disks presented symptoms due to pathogen infection for both genotypes (**Figures 1C,D**). The lesion development was more severe on leaves from the Atenzo cultivar with 36% of important lesions after 96 h, and 72% after 192 h, compared to 18% after 96 h and 22% after 192 h on Gaspard leaves. A treatment with 0.06 mg mL^−1^ RLs delayed pathogenic lesion appearing and reduced their number and size with more than 40% of leaves showing no lesion after 192 h and no distinct and important lesion development for both genotypes. The effect of RLs was stable over time.

### Early and Late Defense Responses Induced by RLs in *B. napus* Foliar Discs

Different defense-inducing activities of RLs were then investigated on Darmor-*bzh* foliar disks from 3 to 4-week-old plants.

**FIGURE 2:**
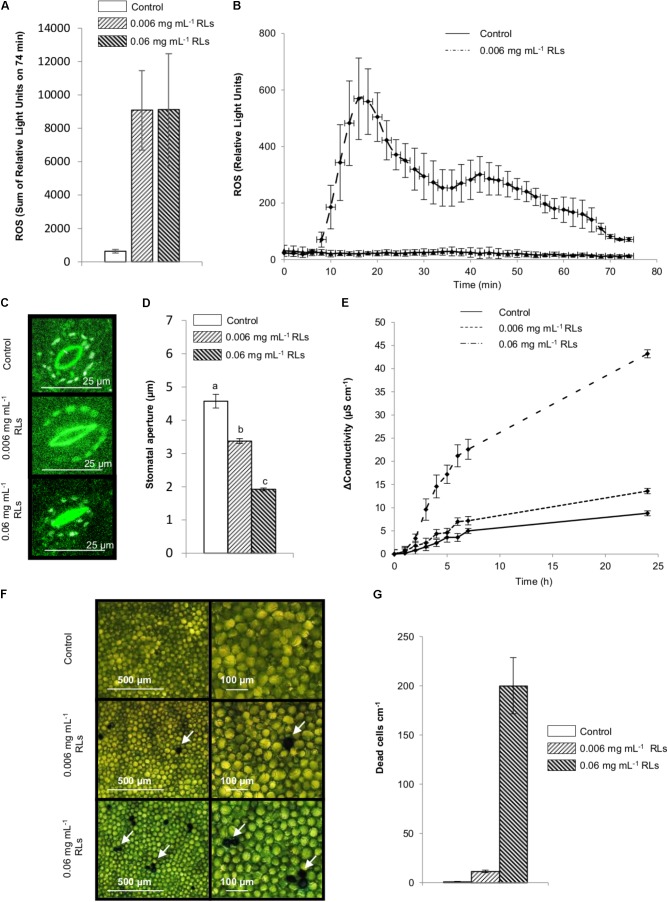
Defense mechanisms induced by RLs in *B. napus* Darmor-*bzh* foliar disks. **(A,B)** Production of ROS by control and RL treated foliar disks. **(A)** Sum of relative light units detected for 74 min. **(B)** Kinetics of ROS production. **(C,D)** Effect of RLs on stomatal closure in foliar disks after 3 h, **(C)** Confocal microscopy observations, **(D)** Stomatal aperture measurements, distinct groups are designated by different letters (one-way ANOVA, α = 0.01). **(E)** Induction of electrolyte leakage by RLs in *B. napus* Darmor-*bzh* foliar disks. **(F)** Evans blue staining of dead cells in leaf foliar disks observed by light microscopy after 24 h. Arrows indicate colored cells **(G)** Number of dead cells per surface of disk after 24 h.

The monitoring of extracellular reactive oxygen species (ROS) accumulation with a luminol-based experiment is classically used for IP activity detection on diverse plants including *B. napus* (Lloyd et al., 2014). To assess the possibility of an early elicitation of *B. napus* by RLs, foliar disks were treated with RL solutions and monitored for the production of ROS over 74 min. A rapid oxidative burst was observed (**Figure 2A**), showing two extrema just before 20 and 40 min with decreasing intensities (**Figure 2B**) and demonstrating the responsiveness of rapeseed to RLs. In the same conditions, the well characterized IP flg22 derived from bacterial flagellin (Lloyd et al., 2014) triggered a single ROS production after 20 min (**Supplementary Figure S3A**).

Stomata are the gate allowing infection for many pathogens like *B. cinerea* (Elad, 1988) and stomatal closure is part of the innate immune response triggered by IPs and micro-organisms in *A. thaliana* (Melotto et al., 2006). To study the effect of RLs on stomata, *B. napus* foliar disks were incubated with RL or control solutions for 3 h before microscopy observations. Stomatal aperture was measured and means of 4.6 ± 0.02 μm for control, 3.4 ± 0.07 μm for 0.006 mg mL^−1^ RL and 1.9 ± 0.04 μm for 0.06 mg mL^−1^ RL solutions were obtained, showing a clear stomatal closure response depending on RL concentration (**Figures 2C,D**).

Plant immune responses induced by elicitors or pathogens can sometimes lead to a hypersensitive response (HR) establishment (Gill et al., 2015). Electrolyte leakage by conductivity measurements is commonly used to quantify changes in membrane permeability triggered by microbes or IPs in plants. It is a classical method to assess HR induction (Johansson et al., 2015). Foliar disks of *B. napus* leaves incubated in RL solutions showed significantly increased conductivity values a few minutes after adding compounds with a dose-dependent effect (**Figure 2E**). Evans blue staining of leaf tissues followed by microscopic observations revealed some individual dead cells or micro-lesions composed of 2–3 cells, 24 h after RL adding, depending on the concentration (**Figures 2F,G**). This suggests that RLs stimulated localized HR-like lesion formation in leaf tissues.

### Defense Gene Induction in *B. napus* Seedlings Upon RL Treatment

To study the capability of RLs to induce a large array of plant defense responses in *B. napus*, we analyzed the expression of early and late defense marker genes. As leaf disks were not an appropriate system to study the expression of belated genes due to RNA degradations, 3-day-old seedlings were challenged with RLs.

**FIGURE 3:**
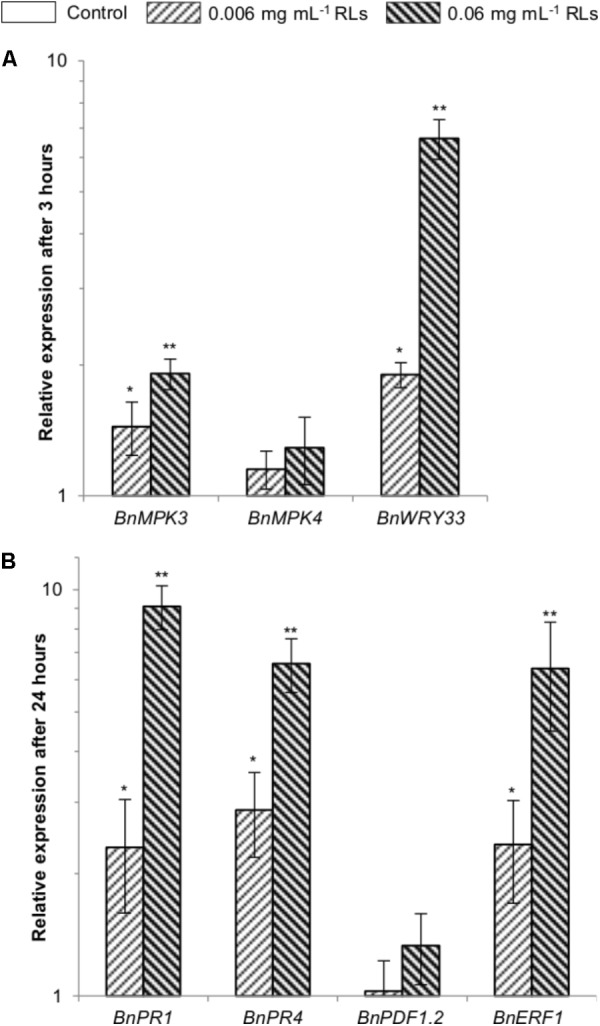
Quantitative qRT-PCR analyzes of defense genes in seedlings treated with RLs. Values were normalized with *ELF5* and related to the control expression at 3 h for *BnMPK3*, *BnMPK4*, and *BnWRKY33* **(A)** and 24 h for *BnPR1*, *BnPR4*, *BnPDF1.2*, and *BnERF1* **(B)**. ^∗^Designates a significant difference as compared to the control condition. ^∗∗^Designates a significant difference as compared to the control condition and to the lower concentration of RLs (one-way ANOVA, α = 0.05).

The expression of the transcription factor *BnWRKY33* as well as *BnMPK3* and *BnMPK4* as signaling MAP Kinase genes, all known as effective early markers of IP responses (Lloyd et al., 2014) was quantified. We observed a light induction of *BnWRKY33* 3 h after addition of 0.006 mg mL^−1^ RLs with a relative expression to the control condition around 1.9 (**Figure 3A**). *BnMPK3, BnMPK4*, and *BnWRKY33* were induced after addition of 0.06 mg mL^−1^ RLs (approximatively 2, 1.5, and 7 times respectively). The expressions of *BnPR1*, *BnPR4*, *BnPDF1.2* were also quantified. These genes are homologous to *AtPR1*, *AtPR4* and *AtPDF1.2*, whose expression is induced in *A. thaliana* RL-treated plants (Sanchez et al., 2012). *AtPR1* is a salicylic acid (SA) specific marker (Lebel et al., 1998). *AtPR4* and *AtPDF1.2* are markers of the cross-talk between jasmonic acid (JA) and ethylene (ET) (Thomma et al., 1998). The expressions of *BnERF1* (marker of ET/JA pathways) (Lorenzo et al., 2003) and *BnMYC2* (marker of JA pathway) (Lorenzo et al., 2004) were also evaluated. Weak inductions of *BnPR1*, *BnPR4* and *BnERF1* were observed 24 h after the 0.006 mg mL^−1^ RL treatment with a relative expression to the control condition around 2 (**Figure 3B**). Inductions around 9 for *BnPR1*, 7 for *BnPR4*, 1.5 for *BnPDF1.2* and 7 for *BnERF1* were measured after addition of 0.06 mg mL^−1^ RLs. No induction of *BnMYC2* was detected in our conditions.

### Callose Synthesis in *B. napus* Leaves Upon RL Spray

**FIGURE 4:**
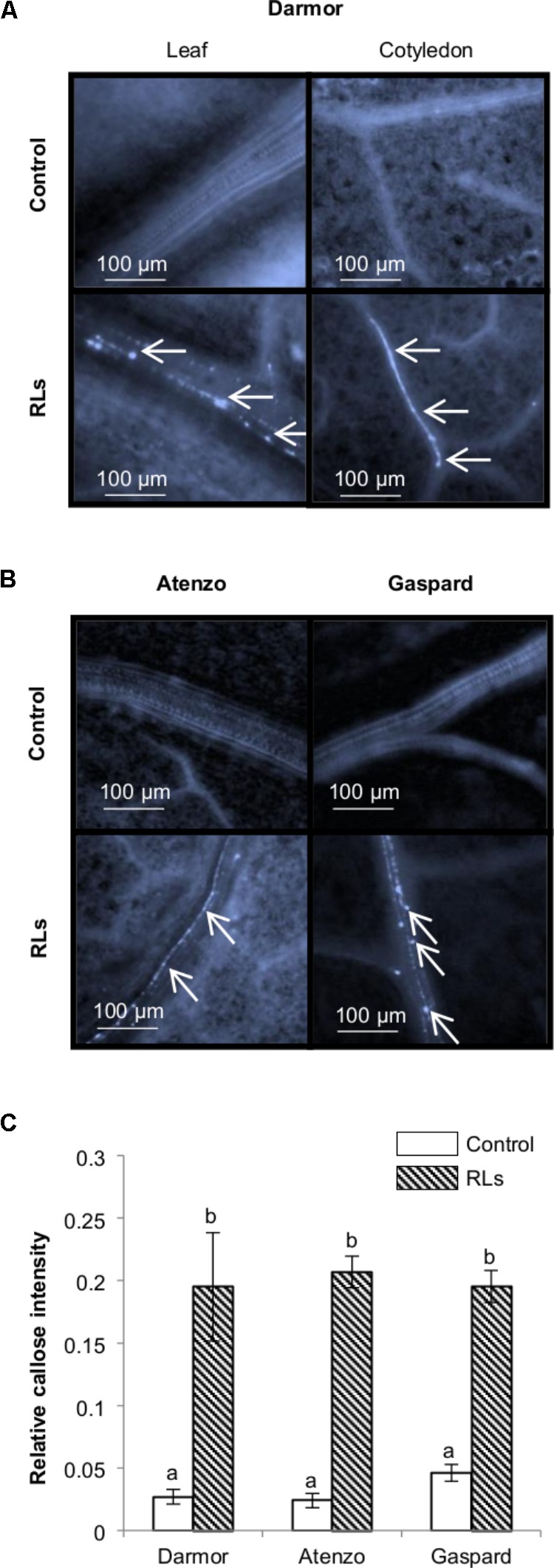
Callose synthesis after a spray treatment of RLs on plants. Fluorescence microscopy observations 24 h after a spray with 0.06 mg mL^−1^ RLs or control solutions on **(A)** Darmor-*bzh*, **(B)** Atenzo and Gaspard. Arrows indicate callose deposits. **(C)** Relative callose intensities. Distinct groups are designated by different letters (one-way ANOVA, α = 0.01).

Callose containing cell-wall appositions are proficient barriers against pathogen invasions and are a matrix for antimicrobial compounds providing targeted delivery of chemical defenses at the cellular sites of attack (Luna et al., 2011). This β-(1,3)-glucan amorphous polymer is a marker of IP-triggered immunity in *A. thaliana* and in *B. napus* (Luna et al., 2011; Lloyd et al., 2014). In order to evaluate the production of this physical protection by RLs, young *B. napus* plants with two well-developed leaves were sprayed with RL or control solutions and the leaves were stained with methyl blue before microscopic observations. This way of treatment was chosen in order to test at the same time the sensitivity of *B. napus* toward RLs in closer conditions to field conditions. RLs were applied at 0.06 mg mL^−1^ as the most efficient and stable concentration used in the protection assays. Visible deposits of callose were observed 24 h after treatments in RL-treated cotyledons and leaves. Callose appositions were particularly visible in vessel elements (**Figure 4A**) as observed for flg22 in same conditions (**Supplementary Figure S3B**). A mean of 0.19 ± 0.04% pixels of pictures, corresponded to callose, was measured in treated leaves or cotyledons, while it represented only 0.03 ± 0.01% on non-treated leaves (**Figure 1C**). Twelve-day-old plants of Atenzo and Gaspard genotypes were also sprayed with RL or control solutions and methyl blue leaf colorations were performed. Clear deposits of callose were visible 24 h after treatment while none were visible in controls (**Figure 4B**). Similarly to what was observed on Darmor-*bzh* genotype, callose appositions were visible in vessel elements. A mean around 0.2% of callose deposits was observed on treated leaves for all cultivars (**Figure 4C**). No significant differences were observed between the responses of Darmor-*bzh*, Atenzo and Gaspard.

### RL Antifungal Activities

**FIGURE 5:**
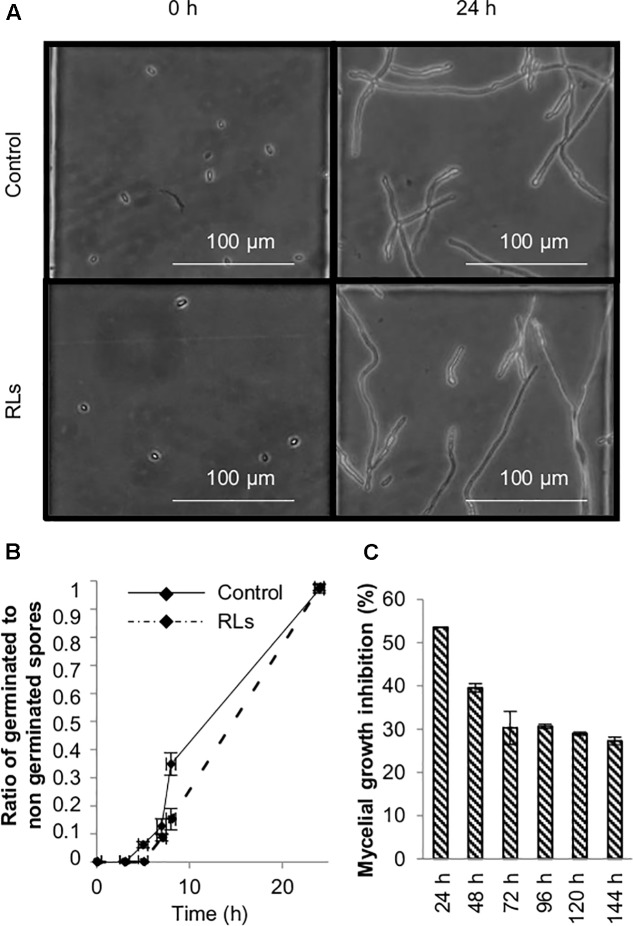
Antifungal effects of RLs. **(A)** Optical microscopy observation of *B. cinerea* conidia germination treated with RLs (0.06 mg mL^−1^). **(B)** Ratio of germinated to non-germinated spores over time. **(C)** *B. cinerea* mycellium growth inhibition in agar medium containing RLs (0.06 mg mL^−1^) as a function of time.

As RLs delay *B. cinerea* conidia germination and inhibit mycelial growth at 0.1 mg mL^−1^ *in vitro* (Varnier et al., 2009), the direct antimicrobial effect of 0.06 mg mL^−1^ RLs was evaluated. Conidia germination and mycelial growth were quantified in appropriate conditions. The ratio of germinated to non-germinated spores was slightly higher in control as compared to the RL-treated condition several hours after treatment. Nevertheless, all conidia germinated in the presence of RLs after 24 h (**Figures 5A,B**). Petri dishes with V8 agar medium supplemented with same amount of RLs were inoculated with agar-mycelium plugs and the mycelium growth inhibition was measured over 6 days. In these conditions, a clear effect was observed with a maximum of 54 ± 2.7% of growth inhibition as compared to the control after 24 h (**Figure 5C**).

### Impact of RL Treatments on *B. napus* Early Development and Chlorophyll Content

**FIGURE 6:**
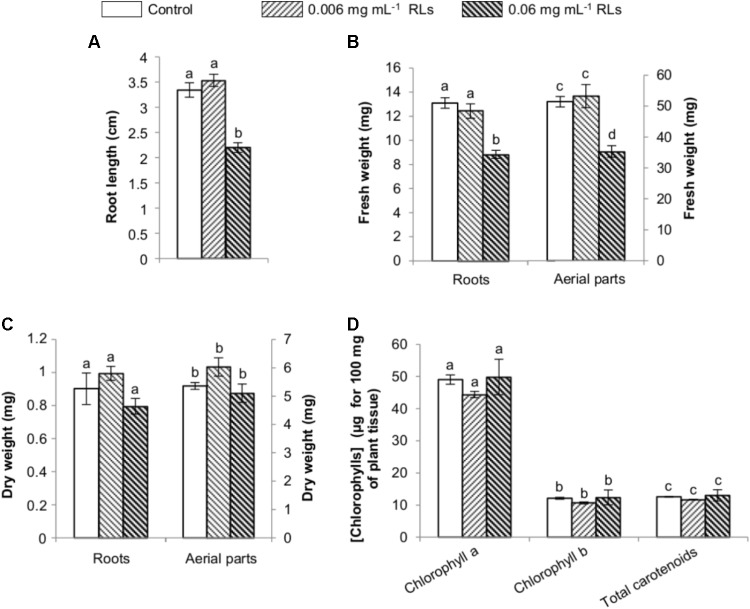
Physiological effects of RL treatments on *in vitro* grown seedlings of *B. napus* Darmor-*bzh* transferred 5 days in RL-added growth medium. **(A)** Root length. **(B)** Fresh weights of roots and aerial parts. **(C)** Dry weights of roots and aerial parts. **(D)** Chlorophyll contents. Distinct groups are designated by different letters (Kruskal–Wallis test, *P* < 0.01).

Elicitors are known to promote defense mechanisms at the expense of growth, and thus the measurement of growth inhibition of seedlings has been proposed to evaluate the activation of an immune response in Arabidopsis or *B. napus* (Vetter et al., 2012). In order to estimate the RL impact on rapeseed early development, the compounds were first added to the agar growth medium of 3-day-old seedlings grown vertically. After 4 days, the root length of the control seedlings was measured (3.34 ± 0.14 cm long). The addition of RLs at 0.006 mg mL^−1^ had no effect on root development. However, 0.06 mg mL^−1^ of RLs inhibited growth by 1.5 times (2.19 ± 0.10 cm long) (**Figure 6A**). This RL concentration also had a repercussion on root and aerial part fresh weights, reducing them approximately 1.5 times. These effects had no consequence on dry weights and chlorophyll contents (**Figures 6B–D**).

**FIGURE 7:**
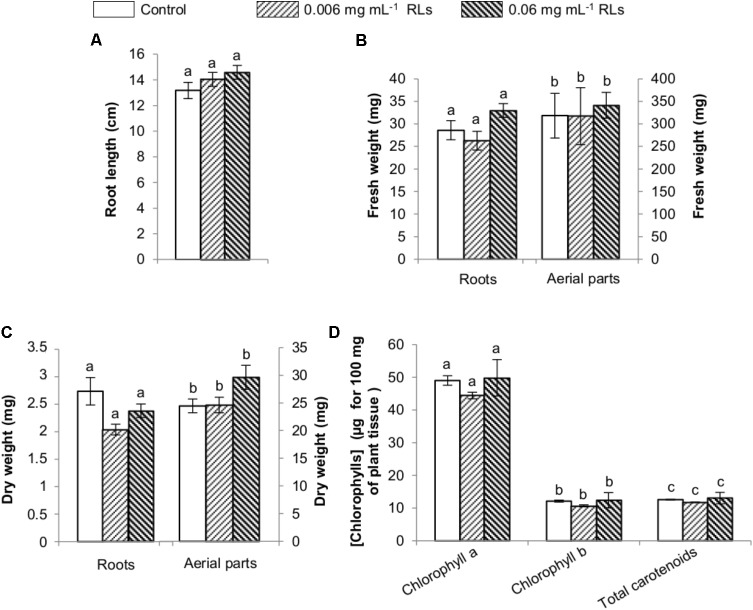
Physiological effects of RL treatments on seedlings of *B. napus* Darmor-*bzh* grown in liquid growth medium and sprayed with RLs. RLs were applied 4 days after transfer in hydroponic conditions and plants harvested 10 days later. **(A)** Root length. **(B)** Fresh weights of roots and aerial parts. **(C)** Dry weights of roots and aerial parts. **(D)** Chlorophyll contents. Distinct groups are designated by different letters (Kruskal–Wallis test, *P* < 0.01).

Rhamnolipids effect was subsequently evaluated on 4-day-old seedlings sprayed with both concentrations and grown in hydroponic conditions. Root length was measured 6 days later and showed no impact of RL treatments (**Figure 7A**). Fresh and dry weights of roots and aerial parts were also compared without any effect (**Figures 7B,C**). Chlorophyll quantifications were performed after treatments and small increases were observed with differences not sufficient to be relevant (**Figure 7D**). No macroscopic symptoms could be monitored on leaves after spraying RLs (**Supplementary Figure S4**).

## Discussion

In this study, the potential of RLs produced by *P. aeruginosa* to protect *B. napus* tissues against the ascomycete *B. cinerea* and to stimulate defense responses were evaluated. Direct applications of 0.006 and 0.06 mg mL^−1^ RLs on foliar disks triggered a resistance against the phytopathogenic fungus by delaying the development of a reduced number of lesions. Furthermore, they were clearly less severe compared to non-treated tissues especially in the case of a 0.06 mg mL^−1^ RL treatment with a stable effect over time. The observation of rapid ROS production, defense gene expression, stomatal closure and electrolyte leakage, clearly demonstrate an early perception of RLs by rapeseed. Late defense responses including the induction of genes involved in defense protein production (PR1, PR4, PDF1.2) and callose deposits were also activated after RL sensing. The direct anti-mycelial growth effect could therefore participate in the delayed and limited evolution of lesions *in planta*, as 30% of mycelial growth inhibition was observed in plate experiments with 0.06 mg mL^−1^ RLs. However, the anti-germinative property could not be responsible for the observed diminution of the number of lesions because all conidia germinated in germination medium after 24 h at the same RL concentration. The beneficial effect of RLs observed on foliar tissues inoculated with *B. cinerea* conidia could therefore combine RL eliciting activities and antifungal effects as it has been suggested for Arabidopsis protection (Sanchez et al., 2012).

We show here for the first time the establishment of plant physical protective mechanisms like callose deposition and stomatal closure triggered by RLs, and involved in the efficient plant protection observed. In Arabidopsis, the transcription of *AtWRKY33* is required for resistance to the fungal pathogen *B. cinerea* (Zheng et al., 2006) and phosphorylation of a transcription factor by MPK3/MPK6 regulates this fungal resistance (Meng et al., 2013). In a same way, the overexpression of *BnMPK4* is known to enhance resistance to *B. cinerea* in *B. napus* (Wang et al., 2009). Hence, the overexpression of *BnWRKY33*, *BnMPK3, BnMPK4* measured here could suggest a role of the corresponding proteins in the protection mechanisms observed. It should be noticed that in Arabidopsis the MPK3/MPK6 cascade activation is necessary to induce the stomatal closure triggered by IPs (Su et al., 2017). The stomatal closure induced by RLs in rapeseed could be linked to the *BnMPK3* overexpression.

Rhamnolipids induce expression of *BnPR1*, *BnPR4* and weakly induce *BnPDF1.2* genes 24 h after treatment as previously described in Arabidopsis with the homologous genes (Sanchez et al., 2012). This suggests that RLs could trigger defense responses dependent on both SA and JA/ET pathways in *B. napus* in connection with the induction of *BnWRKY33* 3 h after treatment. Indeed, in Arabidopsis *AtWRKY33* is an actor of the crosstalk between SA and JA pathway, and its absence has been shown to favor the SA pathway (Birkenbihl et al., 2012). The induction of *BnERF1* by RLs without effect on *BnMYC2* expression suggests that plant defense responses favor the ET pathway.

To date, RL mode of action as antimicrobial compound is not elucidated. Their aggregation behavior could be related to their antifungal activities (Rodrigues et al., 2017). On *Alternaria alternata*, microscopy observations revealed that RLs cause morphological structure alterations in the hyphae, probably due to increased cell permeability with DNA and protein leakages (Yan et al., 2015). More generally, RL inhibition of pathogenic bacteria and fungi have been mainly associated with damage of the microbial cell membrane, reduction of the spore motility and spore collapse (Kim et al., 2000). The biosurfactant property of RLs is then supposed to confer the ability to intercalate into and disrupt the zoospore plasma membrane (Stanghellini and Miller, 1997). It should be noticed that a mycelium growth inhibition around 30% was obtained with 60 μg mL^−1^ RLs on *B. cinerea* in our experiments and with 125 μg mL^−1^ RLs on *A. alternate* (Yan et al., 2015, 2016), confirming that RL antimycelial efficacy is dependent on the pathogen as reviewed by Vatsa et al. (2010).

Size of aggregates that we determined in RL solutions are greater than plant cell wall pores which are around 5 nm (Rondeau-Mouro et al., 2008). Nevertheless, the plant could perceive RLs sprayed on foliar tissues, as shown by callose colorations. Indeed, in this way of treatment there is no wounding of the plant tissues. Thus, neither the foliar wax, particularly thick in rapeseed (Lee and Suh, 2015), nor the cell wall, prevent RL perception. Wax diffusion is probably linked to the biosurfactant properties of RLs and their ability to enhance foliar penetration (Liu et al., 2016).

Such a biphasic ROS profile, as obtained with RLs, is not classically described with other IPs, which induce a transient ROS burst a few minutes after treatment (Lloyd et al., 2014). We should notice that ROS measurements are usually done on durations shorter than 1 h, but such a ROS production kinetic is not obtained with flg22 on rapeseed in our conditions (**Supplementary Figure S3A**). Very recently, a second long-lasting ROS profile has been described in Arabidopsis with lipopolysaccharides, well-known IPs from gram-negative bacteria. However in that case, the second burst reached a peak after 3–10 h and was significantly higher than the first one (Shang-Guan et al., 2018). It seems that ROS response is plant- and elicitor-dependent and the profile described here appears to be a RL signature in rapeseed. The two aggregated forms of RLs in solutions (**Supplementary Figure S1B**) might explain the two extremums observed implicating a possible short perception delay. Nevertheless it should be noted here that local pH and electrolyte variations influence RL aggregation (Sánchez et al., 2007). The RL affinity for membrane lipids (Aranda et al., 2007) should also impact the RL distribution and perception around the plant cell membrane. Currently, the way the plants sense RLs needs further studies.

The replication of experiments on other cultivars than the sequenced Darmor-*bzh* was clearly necessary since responses to elicitors has been shown to be genotype dependent (Lloyd et al., 2014). If Atenzo is a classical oleic cultivar, Gaspard was selected for its high erucic acid content for industrial applications. Since the differences of plant lipid metabolism could influence cuticular wax or cell membrane compositions (Li-Beisson et al., 2013; Lee and Suh, 2015), the lipid elicitor perception could be affected. The efficiency of RL protective effects on those cultivars show the robustness of their perception by grown cultivars.

The concentration of callose depositions in vessel elements, like induced by RLs, was not previously reported for the canonical IP flg22. However, in our experimental conditions, it was also observed on a positive elicitation control (**Supplementary Figure S3B**) showing the importance of the system used to report IP activities. Nevertheless, as RLs trigger defense gene induction when introduced in growth medium of seedlings by root absorption, as we performed in defense gene activation measurements, and induce protections like stomatal closure or callose synthesis when applied on leaves, we show here the robustness of their perception by different parts of *B. napus* plants. RLs could probably be used as well in soil treatments as sprayed on aerial parts in the field as proposed for other elicitors (Thakur et al., 2014). The effect on early development reported here when RLs were applied in growth medium confirmed their IP activity (Lloyd et al., 2014). Though, the study on RL developmental and physiological costs showing no significant reduction of dry weight or chlorophyll content when they were applied by foliar spraying indicates that it is certainly the best way of applying them. RLs could then be a future player of disease biocontrol for rapeseed crop, contributing to the development of sustainable agriculture.

## Author Contributions

NM, CS, and SR conceived and designed the experiments. NM, AF, CB, AD, and GM performed the experiments and different measurements. SD, SC, and CC initiated the protection assays and provided the purified mix of RLs. NM, CS, and SR analyzed the data and wrote the manuscript. All authors helped with drafting the manuscript and approved the final version.

## Conflict of Interest Statement

The authors declare that the research was conducted in the absence of any commercial or financial relationships that could be construed as a potential conflict of interest. The reviewer DP and handling Editor declared their shared affiliation.

## Footnotes

1 http://rsb.info.nih.gov/ij/
