# Supplementary figures and images for: Rhamnolipids From Pseudomonas aeruginosa Are Elicitors Triggering Brassica napus Protection Against Botrytis cinerea Without Physiological Disorders
Source: Front Plant Sci. 2018 Aug 8;9:1170. doi: 10.3389/fpls.2018.01170 (PMC6092566; doi:10.3389/fpls.2018.01170)

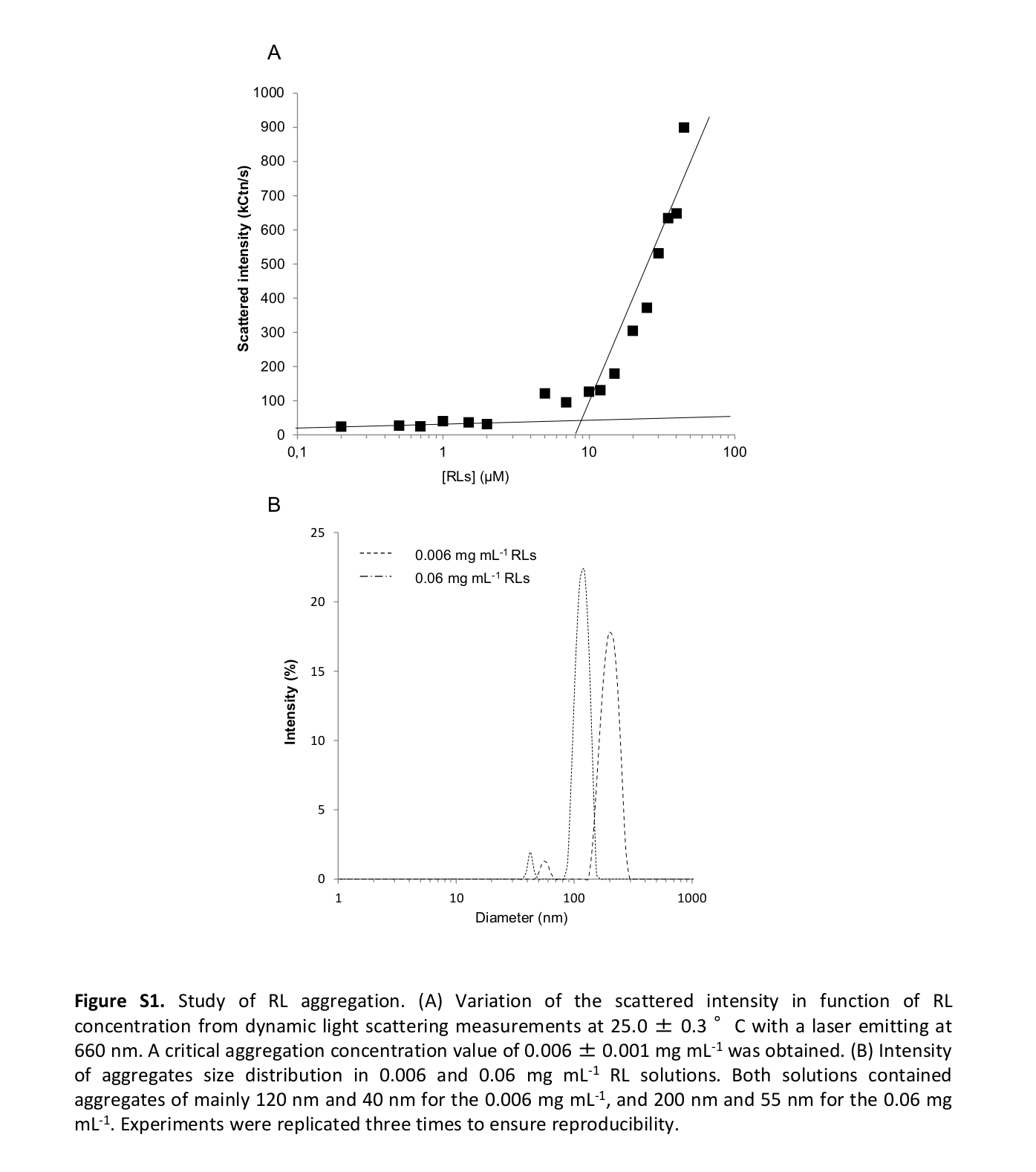

Supplement: Supplementary file 1 [file Image_1.TIF]

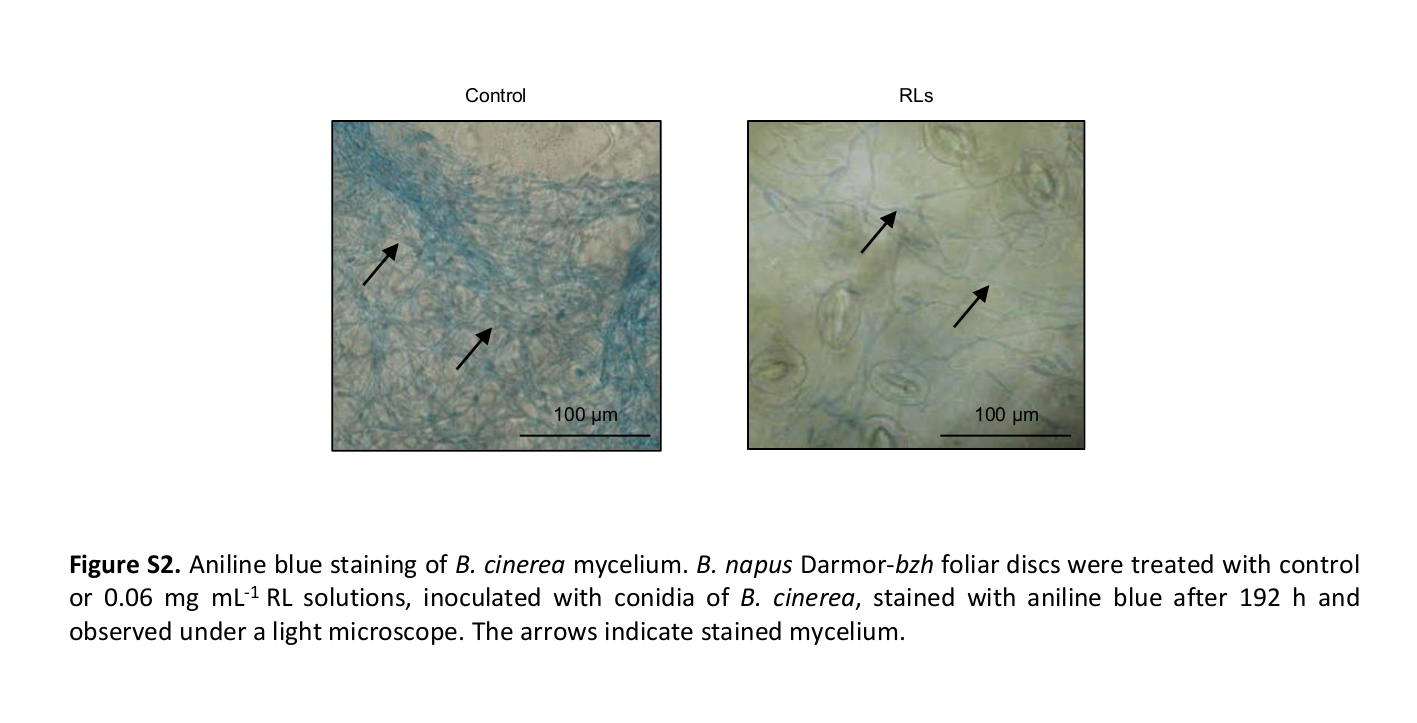

Supplement: Supplementary file 2 [file Image_2.TIF]

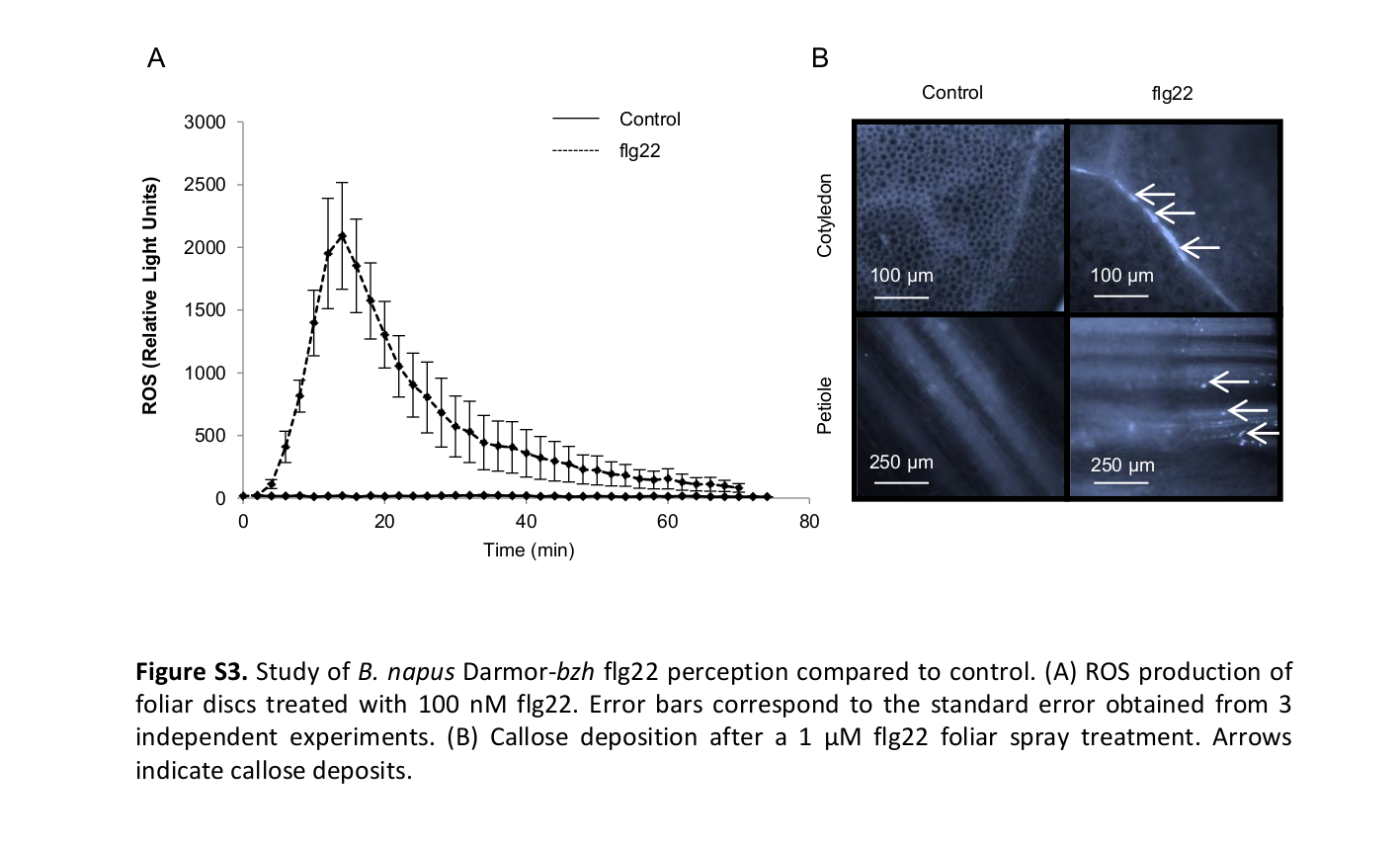

Supplement: Supplementary file 3 [file Image_3.TIF]

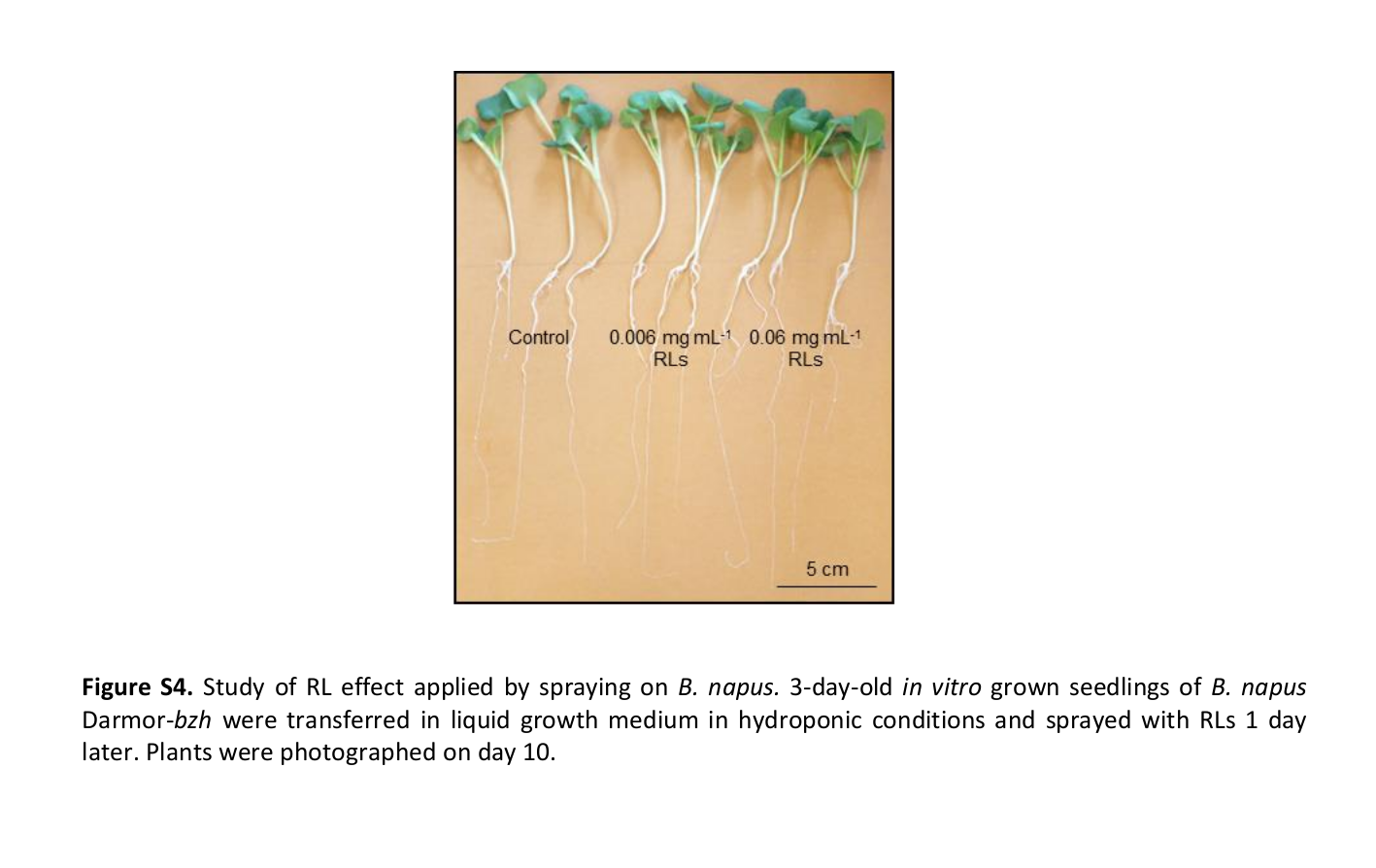

Supplement: Supplementary file 4 [file Image_4.TIF]
